# Hearing Loss in Young Adults: Risk Factors, Mechanisms and Prevention Models

**DOI:** 10.3390/biomedicines13123116

**Published:** 2025-12-18

**Authors:** Razvan Claudiu Fleser, Violeta Necula, Laszlo Peter Ujvary, Andrei Osman, Alexandru Orasan, Alma Aurelia Maniu

**Affiliations:** 1 Department of Otorhinolaryngology, “Iuliu Hatieganu” University of Medicine and Pharmacy, 400347 Cluj Napoca, Romania; dr.fleser@yahoo.com (R.C.F.); violeta.necula@umfcluj.ro (V.N.);; 2 ENT Department, Craiova University of Medicine and Pharmacy, 200349 Craiova, Romania; andrei.osman@umfcv.ro; 3 ENT Department, “Victor Babes” University of Medicine and Pharmacy, 300041 Timisoara, Romania

**Keywords:** hearing loss, young adults, noise-induced hearing loss, ototoxicity, hidden hearing loss, extended high-frequency audiometry, prevention, intervention

## Abstract

Hearing loss is increasingly recognized as a major public health concern among young adults, who are traditionally considered a low-risk group. This narrative review synthesizes recent evidence on risk and aggravating factors of early-onset hearing impairment, including recreational and occupational noise exposure, genetic susceptibility, infections, ototoxic medications, and lifestyle contributors. Pathophysiological mechanisms include cochlear synaptopathy, oxidative stress, excitotoxicity, vascular compromise, and immune-mediated injury. Global Burden of Disease data and World Health Organization reports indicate that more than one billion young people are at risk due to unsafe listening practices. Studies highlight emerging risk factors such as hidden hearing loss, extended high-frequency impairment and associations with COVID-19. Aggravating factors include delayed diagnosis, cumulative exposures and lack of preventive strategies. Early detection via advanced audiological assessments, such as extended high-frequency audiometry, otoacoustic emissions, speech-in-noise testing and auditory brainstem responses, is critical to prevent permanent damage. Public health interventions—particularly safe listening campaigns, early screening and monitoring in high-risk populations—are essential to reduce long-term disability.

## 1. Introduction

The modern era is characterized by an incessant barrage of auditory stimuli, with young individuals often finding themselves immersed in environments filled with various sounds and noises. Global Burden of Disease data and World Health Organization reports indicate that more than one billion young people are at risk due to unsafe listening practices, with approximately 17% of teenagers and 19% of those aged 20–29 already presenting sensorineural hearing loss (SNHL) [1,2,3,4]. In the USA, 1 in 8 individuals aged over 12 years has bilateral hearing loss of varying degrees [5]. Sensorineural hearing loss is the most common type of deafness and is caused by pathology in the inner ear, cochlear nerve, or central nervous system. Audiometric, bone conduction, and air conduction show altered hearing thresholds (reduced below 20 dB), with a difference of less than 15 dB between the two types of conduction at conversational frequencies [6]. Within sensorineural hearing loss is multifactorial and complex, encompassing genetic and environmental factors with substantial occupational contributions, the pathophysiology is represented by dysfunction of the hair cells in the inner ear, cochlear nerve lesions, or central level. Intense noise exposure can lead to temporary or permanent decreases in auditory thresholds. In the case of permanent hearing loss, it is often due to chronic noise exposure (see Table 1). This decrease depends on the intensity and duration of exposure (total amount of noise), as well as individual factors. Individual susceptibility involves factors such as age, sex (men are twice as exposed), exposure history, smoking, diet, and genotype [7]. At the cellular level, there is impairment in the inner ear hair cells (outer and inner), supporting cells, endothelial cells, fibrocytes, spiral ligament, as well as in the dendrites of the cochlear nerve [8].

Hearing loss is traditionally associated with aging, yet its prevalence among young adults has been steadily increasing. Unlike pediatric population and older adults, adolescents and young adults face unique risk exposures, particularly lifestyle and environmental factors, contributing to both temporary and permanent auditory damage. Recent evidence shows that early auditory dysfunction can be detected even in young individuals with normal audiograms. A 2025 study in *Frontiers in Neurology* demonstrated that tinnitus intensity correlates closely with elevated speech-recognition thresholds, indicating early cochlear or neural injury [9]. Early-onset hearing impairment, including tinnitus, can significantly affect academic performance, psychosocial wellbeing, and long-term quality of life which, furthermore, contributes substantially to global disability-adjusted life years (DALYs) and socioeconomic burden. Identifying risk and aggravating factors is critical for prevention, timely intervention, and public health strategies [3,4,10].

Early-onset sensorineural high-frequency hearing loss (HF-SNHL) represents one of the earliest detectable manifestations of cochlear injury in young individuals. High-frequency regions of the cochlea are particularly vulnerable because they are located at the basal turn, where metabolic load, mechanical stress, and exposure to intense acoustic energy are greatest. Experimental and human studies demonstrate that early high-frequency threshold shifts often precede measurable deficits at conversational frequencies and may remain undetected on routine audiometry [11,12,13]. Young adults exposed to recreational noise, personal listening devices, and urban sound environments frequently exhibit subtle but significant elevations in thresholds above 4 kHz, reduced otoacoustic emission amplitudes, and impaired speech perception in noise—even when standard thresholds remain within normal limits [14,15,16]. This pattern reflects early outer hair cell dysfunction and noise-induced cochlear synaptopathy, processes that can evolve into permanent sensorineural hearing loss if exposures persist [14,15,16,17]. Importantly, epidemiological studies indicate that the incidence of high-frequency hearing loss has increased markedly among adolescents and young adults over the last decade, highlighting early HF-SNHL as a modifiable and increasingly prevalent form of auditory impairment requiring targeted preventive strategies [15,17,18,19].

Recent studies report concerning trends. Keppler et al. [14] found that nearly 25% of university students exposed to frequent recreational noise already showed early signs of high-frequency hearing loss. Dillard et al. [18] highlighted that unsafe listening practices are strongly correlated with self-reported tinnitus and diminished speech perception in noise.

Given these trends, young adults should be regarded as a distinct audiological risk group, positioned between pediatric and older adult populations, with specific exposure patterns, mechanisms of injury, and long-term consequences. The current article focuses on summarizing key risk and aggravating factors associated with early-onset hearing loss in young adults, emphasizing the worsening burden in this age group and the ways in which their risk profile differs from that of children and older adults.

## 2. Materials and Methods

### 2.1. Study Design

This review was conducted as a narrative synthesis of recent evidence on predisposing, aggravating factors and potential targeted therapies for hearing loss in young adults. A narrative framework was selected to integrate emerging concepts not yet consolidated within systematic reviews, such as hidden hearing loss and viral-associated auditory dysfunction.

### 2.2. Data Sources and Search Strategy

Literature published between January 2014 and March 2025 was searched in PubMed, Scopus, and Google Scholar. Search strategies combined controlled vocabulary and free-text terms with Boolean operators. Representative queries included: General young adult hearing loss: (“hearing loss” or “noise-induced hearing loss” or “NIHL”) AND (“young adult*” or “18–35 years” or “college student*”) Ototoxicity and medications: (“hearing loss” or “ototoxicity”) AND (“aminoglycoside*” or “chemotherapy” or “cisplatin” or “loop diuretic*”) AND (“young adult*” or “18–35 years”) Emerging mechanisms (hidden hearing loss, viral): (“hidden hearing loss” or “cochlear synaptopathy” or “extended high-frequency audiometry” or “viral infection*” or “COVID-19” or “SARS-CoV-2”) AND (“hearing” or “auditory dysfunction”) Prevention and risk behavior: (“hearing loss prevention” or “noise exposure” or “personal listening device*” or “PLD*” or “music listening”) AND (“young adult*” or “university student*” or “adolescent*”) Targeted therapies and prevention models: (“hearing loss”[Mesh] or “hearing loss” or “noise-induced hearing loss” or “hidden hearing loss”) AND ((“gene therapy” or “genetic susceptibility”) or (“nanoparticle*” or “nanocarrier*” or “nanotechnology”) or (“antioxidant”) or (“anti-inflammatory” or “corticosteroid*” or “dexamethasone” or “steroid therapy”)) AND (“prevention” or “intervention” or “hearing conservation” or “screening” or “early detection” or “extended high-frequency audiometry” or “speech-in-noise”).

Reference lists of included studies and relevant reviews were hand-searched to identify additional sources.

### 2.3. Eligibility Criteria

Publications were eligible if they reported primary data or secondary analyses from clinical investigations, epidemiological surveys, mechanistic or pathophysiological research, or public health reports related to auditory health. Studies focusing on individuals aged 18–35 years were prioritized. Both modifiable (e.g., noise exposure, substance use, listening behaviors) and non-modifiable (e.g., genetic susceptibility, viral infections) factors were considered. Articles not available in English or lacking methodological detail were excluded.

### 2.4. Data Extraction and Synthesis

Data extraction emphasized study design, sample characteristics, exposures, outcomes, and key findings. Evidence was synthesized narratively to highlight convergent results, emerging mechanisms, and knowledge gaps. Special emphasis was placed on risk behaviors and under-recognized mechanisms such as cochlear synaptopathy and viral-related auditory dysfunction.

We declare that to support the writing process, an AI-assisted tool (ChatGPT (GPT-5 series, OpenAI) was used for language refinement, including rephrasing, summarizing, and improving clarity and flow of selected sections. The authors were responsible for the scientific content, interpretation, and final editing, ensuring accuracy and integrity of the work.

## 3. Risk Factors for Hearing Loss

### 3.1. Noise Exposure

Recreational noise exposure, such as prolonged headphone or earbud use, as well as attendance at concerts, nightclubs, and music festivals, has been consistently associated with an increased risk of cochlear damage in high frequency areas of the cochlea—basal turn [14,20]. Similarly, occupational environments, particularly those involving construction, manufacturing, and military service, represent well-documented sources of sustained high-decibel exposure that contribute significantly to the burden of noise-induced auditory pathology even if there are legal regulations that emphasize that these activities must be carried out while using appropriate hearing protection devices [21,22,23,24].

Long-term exposure to noise (>85 dB) involves a multitude of symptoms centered around hearing loss. Initially, patients experience reversible symptoms: temporary hearing loss, tinnitus, vertigo (Tullio phenomenon = a sound-induced vestibular response caused by abnormal communication between the ear’s sound-conducting system and the vestibular system that can occur following perylimphatic fistula, trauma or secondary endolymphatic hydrops), headache, transient otalgia. Subsequently, the symptoms become permanent: progressive hearing loss with the onset of speech understanding disorders (affecting conversational frequencies), tinnitus, distortions, anxiety disorders, decreased productivity, and social isolation [25].

Table 1 summarizes the key differences between temporary threshold shift (TTS) and permanent threshold shift (PTS), highlighting the transition from reversible metabolic cochlear stress to irreversible structural damage associated with prolonged or high-intensity noise exposure.

The pathophysiology of noise-induced hearing loss (NIHL) involves a complex interplay of mechanisms. Repeated or intense acoustic stimulation leads to cumulative outer hair cell (OHC) loss, cochlear synaptopathy, excitotoxic glutamate release, oxidative stress, and mitochondrial dysfunction [26,27,28]. Excessive noise exposure induces calcium overload and activates calpain, ultimately disrupting the PI3K/Akt signaling pathway and triggering hair cell apoptosis [29]. Synaptopathy can develop even in the absence of measurable threshold shifts, a phenomenon often referred to as “hidden hearing loss,” which clinically manifests as difficulty understanding speech in noisy environments [10,13].

The link between OHC loss and the early development of high-frequency sensorineural hearing loss (HF-SNHL) is well established. Because OHCs provide active cochlear amplification and sharpen frequency selectivity, their vulnerability to noise exposure, oxidative stress, and metabolic overload results in preferential degeneration at the basal cochlea, where high frequencies are encoded [8,30]. Even subtle OHC dysfunction can lead to early elevations in thresholds above 4–6 kHz, often before speech-frequency thresholds are affected [8,11,30]. Given that conventional pure-tone audiometry may remain normal in the early stages of OHC injury, the use of otoacoustic emission (OAE) testing is essential. OAEs offer a sensitive, objective measure of OHC integrity and can detect early cochlear damage in young individuals exposed to recreational or occupational noise long before permanent threshold shifts appear [11,31].

Recent investigations have advanced our understanding of these processes. Wang et al., (2025) [32] demonstrated partial synaptic self-repair following noise trauma, thereby identifying a potential therapeutic window for intervention. In a complementary approach, Kitama et al., (2025) [33] reported that calpain inhibitors could preserve cochlear hair cells by maintaining PI3K/Akt signaling integrity. Vasilkov et al., (2023) [17] highlighted the progression of neural degeneration following acoustic trauma, reinforcing the concept that hidden hearing loss may represent a permanent and progressive condition.

These findings underscore that subclinical synaptopathy can produce functional auditory deficits long before conventional audiometric thresholds are affected. Additionally, even though the cochlea is structurally mature, synaptic connections between inner hair cells and auditory nerve fibers remain highly vulnerable in younger individuals exposed to acoustic trauma, resulting in cochlear synaptopathy and hidden hearing loss [13]. This emphasizes the clinical relevance of advanced audiological assessments—such as envelope following responses (EFRs) and auditory brainstem responses (ABRs) with high-rate stimuli—for the early detection of noise-induced auditory dysfunction.

Molnár et al., (2025) [34] identified significant associations between elevated fasting glucose, HbA1c, total cholesterol, triglycerides and LDL levels and subjective tinnitus, suggesting that cochlear injury in young adults may also be exacerbated by metabolic and vascular factors—not only by noise exposure. This underscores the importance of screening for cardiovascular/metabolic risk factors even in otherwise healthy young adults presenting early auditory symptoms [34].

Apart from the auditory system, noise can cause physical and psychological stress, which activates the hypothalamic–pituitary system. This can modulate the physiological activity of hearing. In clinical studies on rats, the group with deficient corticotropin-releasing hormone receptors shows increased susceptibility to noise-induced hearing loss [35,36].

Epidemiological trends regarding hearing loss and auditory risk among adolescents and young adults are summarised in Table 2, highlighting prevalence estimates and key exposure-related factors reported across recent studies.

### 3.2. Genetic Susceptibility

Hearing loss can be broadly classified into syndromic and non-syndromic forms, which differ in etiology, clinical presentation, and genetic background. Syndromic hearing loss refers to auditory impairment that occurs as part of a wider constellation of systemic abnormalities affecting organs such as the eyes, kidneys, thyroid, skin, or craniofacial structures. Examples include Usher syndrome, Pendred syndrome, Waardenburg syndrome, and Alport syndrome, in which hearing loss is accompanied by visual, metabolic, or renal manifestations [28,42,43]. In contrast, non-syndromic hearing loss presents as an isolated auditory deficit without additional systemic findings and accounts for approximately 70–80% of hereditary hearing loss cases [44,45]. Non-syndromic forms are most commonly associated with mutations in genes encoding ion channels, gap-junction proteins, or structural components of the cochlea—such as GJB2 (connexin 26), TECTA, OTOF, and MYO7A [44,45].

Several studies have revealed that specific genetic and epigenetic characteristics can heighten susceptibility to acoustic trauma in animal models of hearing loss. Transgenic mice expressing genes associated with age-related hearing loss, such as Ahl1 (Cdh23735A > G) in C57BL/6J mice, demonstrate increased vulnerability to further hearing deterioration induced by noise exposure. Mice lacking Ahl1 (e.g., 129/SvEv, Cast/Ei, and MOLF/Ei) exhibit reduced susceptibility to acoustic injury. Research on knockout mice has identified pathways involving cochlear structures, oxidative stress, potassium recycling (essential for sensory transduction), and heat shock proteins (HSPs) that augment the inner ear’s susceptibility to NIHL. Among the mouse genes implicated in these pathways are *Cdh23*, *Pmca2*, *Sod1*, *Gpx1*, *Trpv4*, *Vasp*, *Hsf1*, and *mdx* [25].

Genes involved in pathways related to oxidative stress, potassium recycling, and heat shock proteins (HSPs) have been linked to noise-induced hearing loss (NIHL) in humans. Within the oxidative stress pathway genes, polymorphisms of GSTM1, PON2, SOD2 and CAT have been associated with NIHL and CAT was independently validated in Swedish and Polish populations. Ten genes in the potassium recycling pathway, including GJB1, GJB2, GJB3, GJB4, GJB6, KCNJ10, KCNQ1, KCNQ4, KCNE1 and SLC12A2 have known or suspected associations with both syndromic and non-syndromic hearing loss. An analysis of 35 single-nucleotide polymorphisms (SNPs) in these genes from a Swedish population revealed associations between KCNE1, KCNQ1, and KCNQ4 with NIHL [46]. Reduced KCNQ4 channel activity, which normally supports potassium recycling and maintains ionic homeostasis in the inner ear, has been linked to genetic, noise-induced, and age-related forms of hearing loss [47]. A Polish study replicating the findings reported associations between polymorphisms in GJB1, GJB2, GJB4, KCNJ10, and KCNQ1 with NIHL [25].

GJB2 mutations, which encode the gap-junction protein connexin 26, represent the most common genetic cause of congenital and early-onset non-syndromic sensorineural hearing loss worldwide. The majority of GJB2-associated cases follow an autosomal recessive inheritance pattern, meaning affected individuals inherit biallelic pathogenic variants—one from each carrier parent—while heterozygous carriers typically have normal hearing. This mode of inheritance explains the high prevalence of GJB2-related hearing loss in newborn screening programs despite parents being asymptomatic. Although less frequently, some GJB2 variants can also present with autosomal dominant inheritance and a variable phenotype, including progressive or milder high-frequency loss. Clarifying this pattern is important for counseling, recurrence risk estimation, and designing effective early-detection strategies [42,48,49,50].

Mutations in GJB2 (connexin 26), mitochondrial DNA variants, and other deafness-associated loci elevate vulnerability to early-onset sensorineural hearing loss [51]. Genetic predispositions amplify susceptibility to noise, ototoxic drugs, and sudden sensorineural hearing loss (SSNHL) by compromising cochlear resilience [52]. Polymorphisms within the heat-shock protein 70 (HSP70) gene family, particularly *rs1061581* and *rs2227956*, have emerged as genetic susceptibility markers for noise-induced hearing loss (NIHL), especially in Caucasian male cohorts, as demonstrated by a robust meta-analysis (Lei et al., 2017) [53]. HSPs play crucial roles in synthesizing, folding, assembling, and transporting various proteins within cells. Their expression increases during oxidative or other stress, including exposure to noise, potentially offering protection against cochlear damage by stabilizing stereocilia or regulating enzymes. Three polymorphisms in HSP70 genes—HSP70-1, HSP70-2, and HSP70-hom—have been linked to susceptibility to NIHL [16].

Several dominantly inherited types of hearing loss present in adulthood, including DFNA9, which is caused by COCH missense variants. DFNA9 typically manifests as progressive high-frequency hearing loss with possible vestibular dysfunction, while recessive COCH mutations lead to early, severe loss (DFNB110). COCH encodes cochlin, an extracellular matrix protein in the inner ear, whose LCCL domain is thought to contribute to local innate immunity, suggesting increased susceptibility to infections in DFNA9 [54]. These features clinically overlap with Ménière’s disease. This reinforces the concept that, beyond environmental and idiopathic mechanisms, specific genetic alterations can predispose individuals to early or atypical presentations of Ménière-like symptoms, including fluctuating sensorineural hearing loss, vertigo, and tinnitus [55].

Genetic factors also influence the risk of hearing loss associated with ototoxic medications and structural anomalies. Individuals harboring mitochondrial MT-RNR1 variants such as m.1555A > G, m.1494C > T and m.1095T > C are at markedly increased risk of irreversible hearing loss even at therapeutic doses of aminoglycoside antibiotics [56].

Structural genetic syndromes like SLC26A4-related enlarged vestibular aqueduct (EVA) highlight gene–environment interplay: individuals with EVA are predisposed to fluctuating or sudden hearing loss triggered by minor head trauma or changes in pressure [57].

### 3.3. Infections and Systemic Illnesses

Viral infections, including measles, mumps, and rubella, are well-documented causes of cochlear injury, with multiple pathogenic mechanisms implicated such as direct viral neurotropism, cytokine-mediated inflammatory damage, microvascular thrombosis, and secondary autoimmune responses. More recently, attention has focused on the auditory consequences of SARS-CoV-2 infection. In a large cohort analysis, Kim et al., (2024) [58] demonstrated that young adults with a history of COVID-19 had a 3.4-fold increased risk of developing hearing loss and a 3.5-fold increased risk of SSNHL compared with non-infected controls. Experimental models further support the plausibility of viral entry into the inner ear: inner ear organoid studies have shown that SARS-CoV-2 may infect cochlear and vestibular cells directly, potentially leading to structural and functional compromise [59].

Clinically, electrophysiological investigations also indicate central auditory involvement; a recent 2025 study identified auditory brainstem response (ABR) abnormalities in post-COVID patients, suggesting that viral damage may extend beyond the cochlea into higher auditory pathways. The alterations described include prolonged absolute latencies of waves I, III and V, increased inter-peak intervals (I–III, III–V, I–V), and reduced wave V amplitude, findings consistent with impaired neural conduction along the auditory brainstem pathways [60,61,62].

Bacterial infections likewise remain a critical cause of irreversible auditory morbidity. Bacterial meningitis in particular carries a high risk of profound sensorineural hearing loss, largely due to the combination of intense labyrinthine inflammation and subsequent ossification of the cochlea (sometimes beginning 2 weeks after meningitis infection), processes that may rapidly eliminate the potential for cochlear implantation if not recognized and managed in a right time [63,64,65]. Bacterial involvement of the inner ear may occur through descending infection in meningitis—where inflammation extends into the cochlea or through ascending spread from acute or chronic middle ear infection, which can also progress to intracranial complications such as meningitis [64,66]. Additionally, systemic infection such as sepsis may further facilitate bacterial dissemination to both the middle and inner ear [67].

### 3.4. Ototoxic Agents

A variety of pharmacological agents and recreational substances have been implicated in cochlear injury through distinct but often convergent mechanisms.

Aminoglycoside antibiotics (primarily cochleotoxic—kanamycin, amikacin, neomycin) and cisplatin are well-recognized ototoxic agents, inducing irreversible hair cell apoptosis primarily via excessive reactive oxygen species (ROS) generation and mitochondrial dysfunction [68,69,70,71].

Although loop diuretics are not usually prescribed for this age group, when used, they exert their effects by altering endolymph composition, which may result in reversible auditory threshold shifts but, in some cases, can also lead to permanent sensorineural damage [68]. Nonsteroidal anti-inflammatory drugs (NSAIDs) represent a pharmacological group frequently used by young adults, which has been associated with reversible SNHL; consequently, limiting excessive self-medication and polypharmacy remains essential [72]. NSAID-induced sensorineural hearing loss results from reversible inhibition of outer hair cell electromotility, reduced cochlear blood flow due to prostaglandin suppression, disturbances in cochlear ion homeostasis, and central auditory hyperexcitability—mechanisms that collectively produce temporary high-frequency threshold elevations and tinnitus [73,74,75].

Anti-epileptic medications are believed to act on multiple levels of the auditory system. Compounds including valproate, vigabatrin, and gabapentin can interfere with the electromagnetic conduction of hair cells and afferent neurons through their effects on GABA (Gamma-Aminobutyric Acid) receptors [72,76].

Beyond prescribed medications, recreational drugs such as cocaine, methamphetamine, and 3,4-methylenedioxymethamphetamine (MDMA) compromise cochlear microcirculation and intensify oxidative stress, thereby exacerbating the risk of noise- or drug-related auditory injury [77].

The risk of ototoxic hearing loss does not operate in isolation. Genetic predisposition can interact with environmental stressors such as noise exposure, amplifying the extent of cochlear injury and accelerating the onset of clinically significant deficits [28,51,52,68]. This cumulative vulnerability highlights the necessity of proactive monitoring strategies. Preventive approaches should include baseline and serial audiometric testing, complemented by objective measures such as otoacoustic emissions (OAEs), particularly in individuals with known genetic susceptibility or anticipated high cumulative exposure to ototoxic agents.

### 3.5. Lifestyle Factors

Several lifestyle factors have been implicated in modulating susceptibility to auditory injury through mechanisms involving oxidative stress, vascular compromise, and impaired neural regulation. Cigarette smoking is consistently associated with heightened oxidative stress and diminished cochlear perfusion, thereby potentiating vulnerability to both noise and ototoxic insults [77]. Alcohol consumption exerts a more complex influence. While chronic excessive intake and malnutrition weaken endogenous antioxidant defenses and reduce cochlear resilience, moderate alcohol use has been reported to exert neutral or even protective effects, possibly mediated through improved cardiovascular function [77]. Acute alcohol exposure, however, can transiently impair auditory thresholds, particularly at lower frequencies, an effect that is typically reversible. This bidirectional association has been described as a U-shaped relationship between alcohol intake and hearing loss risk [77].

Psychological stress and circadian disruption have emerged as relevant modulators of auditory health. Experimental and epidemiological evidence indicates that chronic stress and sleep deprivation impair neurovascular homeostasis, reduce inner ear perfusion, and exacerbate susceptibility to acoustic trauma and other forms of cochlear injury [78]. These findings emphasize that lifestyle-related factors, while often considered secondary, play a substantial role in shaping both the risk and progression of sensorineural hearing loss.

### 3.6. Psychosocial and Sociodemographic Factors

Limited awareness, peer norms, and inadequate access to ear protection or volume-limiting technology perpetuate unsafe listening behaviors. Socioeconomic disparities further delay diagnosis and limit preventative measures [18,79].

## 4. Aggravating Factors of Hearing Loss

Evidence suggests that young adults are especially susceptible to the compounded effects of environmental and lifestyle determinants of auditory health. The lack of protective strategies in both recreational and occupational settings remains a major concern, as individuals frequently underestimate cumulative risk from high-intensity exposures [79].

A recent experimental study has shown that when present together, ototoxic drugs and noise exposure exert a synergistic ototoxic effect, characterized by intensified oxidative stress, an amplified inflammatory cytokine response within the cochlea, and increased expression of apoptosis-related proteins, culminating in more pronounced morphological and functional injury [80].

This cumulative effect is particularly significant in the young adult population, in whom recreational headphone use, loud volume, recreational drug use and other lifestyle factors are more often superposed compared to other age groups [81,82]. Recent studies show a growing tendency of drug use in the young population together with the already well documented tendency of above safety threshold listening to music. Among Canadians aged 20–24, cocaine use rose to 9% in 2021, tripling since 2013, while ecstasy use increased to 5.5% from 3.1% in 2017. Rates are substantially higher in the EDM scene, where a 2022 New York City study found past-year use of cocaine (22%), ecstasy (19%), and ketamine (11%) among event attendees [82,83].

Specifically, in young adults, recreational drug use frequently co-occurs with high-intensity noise exposure in settings such as clubs, concerts, and festivals, creating a synergistic increase in cochlear vulnerability. Clinical and experimental studies have shown that cocaine, MDMA, amphetamines, and cannabis potentiate noise-induced oxidative stress, vasoconstriction, and synaptic dysfunction, amplifying the risk of tinnitus, temporary threshold shifts, and early high-frequency sensorineural hearing loss in young adults [84,85,86].

Furthermore, the delayed recognition of hidden or extended high-frequency hearing loss often allows subclinical damage to progress to irreversible deficits before patients seek medical evaluation [87,88].

So individual risk factors are compounded by synergistic exposures, in which concurrent influences such as excessive noise, ototoxic pharmacotherapy and underlying genetic susceptibility act in combination to accelerate cochlear degeneration [13,26].

Diagnostic limitations contribute to missed opportunities for early detection. The underuse of advanced audiological tools—including extended high-frequency audiometry, otoacoustic emissions (OAEs), speech-in-noise testing, and auditory brainstem responses (ABRs)—restricts the ability to identify auditory dysfunction at its earliest stages [60,89,90]. We believe that current standards in audiological testing and prevention do not factor in new lifestyle-related risk factors for this specific population. Thus, a novel multi tier approach system would be beneficial for advancing precision prevention and mitigating the long-term burden of hearing loss. Many individuals present late for evaluation because they are unaware of the importance of early recognition and timely assessment of auditory symptoms. Improving patient education is essential for early detection and prevention of progressive hearing loss. In addition, patients often continue exposure despite early warning symptoms with young adults more predisposed on minimizing or dismissing the signs.

## 5. Assessment of Hearing Loss

Conventional pure-tone audiometry (PTA), spanning 0.125–8 kHz, remains the gold standard for detecting clinically overt sensorineural hearing loss. However, it lacks the sensitivity to identify subclinical or “hidden” pathologies, particularly in patients who report functional hearing difficulties despite normal audiometric thresholds and also lacks objectivity which is a limitation in some age groups (children) or categories of patients (psychiatric conditions) [87,88]. To address the limitation of identifying subclinical pathologies, extended high-frequency (EHF) audiometry—ranging from 9 to 20 kHz—has emerged as a valuable tool, enabling the detection of early cochlear changes that precede measurable deficits in the conventional frequency range [13,89].

Otoacoustic emissions (OAEs) provide an objective measure of outer hair cell integrity (cochlear testing) and are capable of revealing dysfunction before threshold shifts appear on standard audiometry [10,26]. Speech-in-noise testing is increasingly recognized as a critical complement, as it captures functional deficits in auditory processing under ecologically valid listening conditions, which are often missed by traditional audiometry [87,88]. Auditory brainstem responses (ABRs) objectively evaluates the integrity of the auditory pathway from the cochlea to the brainstem by recording neural electrical activity in response to sound stimuli, allowing identification of threshold sensitivity, neural conduction delays, and retrocochlear pathology [91]. Recent evidence has demonstrated their utility in detecting central auditory pathway alterations following viral infections such as SARS-Cov-2 [60].

Key distinctions in the aetiology, risk factors, pathophysiological mechanisms, and clinical presentation of hearing loss across age groups are summarised in Table 3, which compares paediatric, young adult, and older adult populations.

## 6. Targeted Interventions and Prevention Modalities

### 6.1. Potential Targeted Interventions

Recent research has focused on gene- and molecule-based interventions. Gene therapy trials are ongoing for congenital forms of hearing loss caused by single-gene mutations (e.g., OTOF, GJB2), with early results suggesting partial restoration of auditory function. Neurotrophins and growth factors, such as brain-derived neurotrophic factor (BDNF), neurotrophin-3 (NT-3) and insulin-like growth factor-1 (IGF-1) are being investigated for their potential to protect or regenerate spiral ganglion neurons and hair cells. In addition, epigenetic modulators including histone deacetylase (HDAC) inhibitors have shown preclinical promise in reducing hair cell apoptosis after ototoxic or noise-induced injury [92].

Activation of the KCNQ4 potassium channel has been proposed as a targeted therapeutic strategy in genetic, noise-induced, and age-related hearing loss. Molecules such as retigabine and its derivatives, as well as novel compounds (zinc pyrithione, ML213, acrylamide S-1), can restore channel function and protect outer hair cells; however, the development of selective and efficient in vivo activators remains a priority for clinical translation [47].

Systematic genotype–phenotype correlations in DFNA9 highlight that specific COCH variants, particularly those affecting the LCCL domain, are associated with faster hearing loss progression. These insights provide a crucial framework for designing variant-specific targeted interventions, including emerging genetic therapies, and for defining appropriate timing and clinical endpoints in future trials [54].

Several pharmacological strategies aim to mitigate inner ear damage. Antioxidants and free-radical scavengers (e.g., N-acetylcysteine, D-methionine, coenzyme Q10) have been explored for the prevention of cisplatin- and noise-induced hearing loss, though clinical evidence remains mixed. Anti-inflammatory agents represent another approach; while corticosteroids remain the clinical standard, novel molecules such as TNF-α and JNK pathway inhibitors are under investigation. Furthermore, iron chelators (e.g., deferoxamine) have been proposed to attenuate cisplatin-induced ototoxicity by reducing reactive oxygen species formation [93].

Local delivery techniques have been developed to maximize drug concentration in the cochlea while minimizing systemic exposure. Intra-tympanic and round-window administration of steroids, small interfering RNAs (siRNA) and growth factors are being studied in this context. Additionally, nanoparticle-based carriers represent an emerging strategy for the targeted release of protective agents directly to cochlear hair cells [94]. Several classes of nanocarriers have been investigated, including lipid-based nanoparticles (lipid core nanocapsules, solid lipid nanoparticles, phospholipid nanoparticles), polymeric nanoparticles (polymersomes, PLGA, chitosan), and metallic or inorganic nanostructures (silver, gold, silica, and superparamagnetic iron oxide nanoparticles). These delivery platforms can cross cochlear barriers, provide sustained release, and enhance therapeutic efficacy while minimizing systemic toxicity. Recent studies conducted at our research center (UMF ‘Iuliu Hațieganu’ Cluj-Napoca) highlight the potential of such nanocarriers to overcome current limitations of systemic and intratympanic therapies, paving the way for future clinical translation in sensorineural hearing loss [95,96].

A biopolymer–lipid hybrid microcarrier designed for transmembrane delivery of dexamethasone to the inner ear has been studied. This platform achieved high encapsulation efficiency, provided sustained release for up to six days, and protected auditory cells from cisplatin-induced toxicity in vitro [96].

Novel regenerative strategies focus on restoring lost sensory cells. Atoh1 gene activation has been investigated to induce supporting cell transdifferentiation into hair cells; while early clinical trials (e.g., GenVec/Novartis CGF166) demonstrated safety, efficacy has so far been limited. Stem cell-based therapies are also under exploration, with the goal of repopulating damaged hair cells and auditory neurons [97].

The rise of precision medicine allows for stratification of at-risk individuals. Genetic screening for susceptibility, such as mitochondrial 12S rRNA mutations that cause a predisposition to aminoglycoside ototoxicity, enables tailored preventive strategies. By identifying genetically vulnerable populations, interventions can be more effectively targeted to reduce long-term auditory decline [98].

Until emerging targeted therapies become clinically available, cochlear implantation remains the most effective rehabilitative option for individuals meeting established audiological criteria, providing significant improvements in sound perception, speech understanding, and overall quality of life [99,100]. At the same time, for individuals who do not fulfill the audiological criteria for cochlear implantation, appropriately fitted hearing aids represent the primary rehabilitative approach.

### 6.2. Preventive Models

While targeted interventions such as gene therapy, regenerative approaches, and pharmacological otoprotection hold considerable promise, most remain in early experimental or preclinical stages, with limited translation into routine clinical practice. Consequently, in young adults—in whom much of the auditory risk is driven by behavioral and environmental exposures—prevention modalities currently represent the most effective and immediately applicable strategy to mitigate long-term hearing decline. Shifting focus from therapeutic innovation to preventive frameworks is therefore essential for reducing the global burden of hearing loss in this age group.

Preventive strategies for young adults focus primarily on modifiable behaviors, particularly those related to recreational noise exposure. Educational campaigns such as the WHO “Make Listening Safe” initiative emphasize the importance of safe listening practices when using personal music players and attending concerts or clubs. Promoting simple guidelines, including the “60–60 rule” (listening at ≤60% of maximum volume for ≤60 min per day), has proven effective in raising awareness and reducing cumulative auditory risk. University-based hearing conservation programs further highlight the importance of early education in shaping lifelong listening habits [4].

At the population level, regulatory interventions provide a framework for prevention. The European Union, for example, has mandated that personal listening devices be set to a default maximum of 85 dB, while still allowing users to override settings with explicit warnings. Occupational safety regulations (e.g., OSHA in the USA, EU noise directives) mandate regular monitoring, provision of hearing protection and exposure limits for workers in high-risk environments such as music venues, military service, or industrial settings. These measures extend beyond occupational health and increasingly target leisure noise, reflecting the shift in risk burden toward younger populations [101,102].

Early identification of auditory changes is central to prevention. Extended high-frequency audiometry and otoacoustic emissions (OAEs) allow detection of subclinical cochlear damage before standard pure-tone audiometry reveals deficits. Integrating regular hearing checks into school or university health programs, as well as the development of smartphone-based applications that monitor daily sound exposure, represent scalable approaches for early detection. Such tools not only enable timely intervention but also reinforce risk awareness in tech-savvy young populations [103].

Additional screening modalities can enhance early detection of auditory dysfunction in young adults. Auditory brainstem responses (ABR) and electrocochleography (EcochG) provide objective measures of cochlear synaptopathy, with reduced Wave I amplitude or elevated SP/AP ratios reported as early biomarkers of hidden hearing loss, even when pure-tone thresholds are normal [104]. Speech-in-noise testing, particularly in combination with electrophysiology, captures suprathreshold deficits relevant to real-life communication in young populations [104]. Recent advances include AI-based automation of digits-in-noise (DIN) tests, which significantly reduce cost and development time, making community-wide or online screening feasible [105]. Furthermore, smartphone-based applications, such as the Johns Hopkins “Hearing Number” app, enable self-assessment of hearing thresholds and may increase awareness and engagement in preventive practices among adolescents and young adults [106]. Together, these approaches extend beyond conventional audiometry and open avenues for personalized, accessible and scalable early diagnostic strategies.

Table 4 provides an overview of the major risk factors associated with hearing loss in young adults, highlighting their main characteristics.

## 7. Discussions

This review highlights that hearing loss in young adults is a growing but underrecognized global health issue. Our synthesis confirms that in this population, SNHL is inherently multifactorial, as recreational and occupational noise exposure remains the most prominent modifiable risk factor (along other lifestyle factors such as drug consumption), consistent with WHO estimates that over one billion young people are at risk due to unsafe listening practices [1,2,3,4]. Emerging evidence on hidden hearing loss and extended high-frequency deficits indicates that conventional audiometry underestimates the true burden of early auditory dysfunction [9,26,89]. Mechanistic studies further demonstrate that synaptic injury, excitotoxicity, oxidative stress, and mitochondrial dysfunction underlie much of the observed pathology [8,27,32].

The genetic susceptibility literature suggests that individuals with GJB2 or mitochondrial mutations are more vulnerable to environmental and pharmacological insults, which aligns with findings in cancer survivors exposed to cisplatin [28,52,56]. Infections, particularly COVID-19, have introduced a new dimension to early hearing loss risk, with both peripheral and central auditory involvement documented through auditory brainstem response abnormalities [58,59,60]. This warrants longitudinal studies to determine long-term outcomes in post-viral populations.

Ototoxic agents continue to pose risks in both clinical and recreational contexts. Aminoglycosides, cisplatin, and certain recreational substances act via overlapping oxidative and vascular pathways [69,70,76]. Lifestyle contributors such as smoking, poor diet, alcohol use and chronic stress further amplify susceptibility by weakening cochlear defense systems [77,78].

Aggravating factors such as delayed diagnosis and cumulative exposures accelerate progression from subclinical to irreversible impairment [80,81]. The underuse of extended high-frequency audiometry, OAEs, and speech-in-noise testing remains a major diagnostic gap [87,88,89]. Recent work with ABR has shown potential for detecting central auditory sequelae, expanding the diagnostic toolkit [80,81].

From a public health perspective, safe listening campaigns have shown promise, but their uptake is uneven across regions. Young adults often underestimate their risk and socioeconomic barriers limit access to protective measures and preventive screening [18,79]. More targeted interventions in high-risk groups—such as students, military personnel, and construction workers—are urgently needed.

Limitations: As a narrative review, our synthesis cannot exclude publication bias, and the heterogeneity of study designs limits direct comparability. More longitudinal cohort studies and mechanistic human trials are required to clarify causal pathways and evaluate interventions.

Future Directions: Research should focus on (1) developing objective biomarkers of hidden hearing loss (e.g., envelope following responses, high-rate ABRs), (2) gene- and molecule-based interventions (3) testing pharmacological protective agents such as antioxidants and calpain inhibitors, (4) integrating extended high-frequency audiometry and otoacoustic emissions into routine screenings for high-risk populations (university students, military personnel, oncology patients), and (5) implementing population-level preventive programs through schools, universities, and occupational health systems.

## 8. Conclusions

Hearing loss in young adults is a burgeoning global health concern propelled by modifiable and non-modifiable risk factors. Mechanisms such as synaptopathy, oxidative stress, excitotoxicity, and vascular or immune-mediated injury underlie early-onset impairment. Advanced diagnostic tools—extended high-frequency audiometry, OAEs, speech-in-noise testing, and ABRs—are essential for early detection. Public health efforts must focus on safe listening campaigns, integration of screening in educational and occupational settings, and regulatory protections. Innovations such as genetic profiling and pharmacologic protectants (e.g., calpain inhibitors) offer promising prevention paths. A coordinated effort across research, clinical practice, and policy is vital to preserve hearing health and prevent lifelong disability among young adults.

## Figures and Tables

**Table 1:** Comparison between Temporary Threshold Shift (TTS) and Permanent Threshold Shift (PTS).

Feature	Temporary Threshold Shift (TTS)	Permanent Threshold Shift (PTS)
Definition	Reversible elevation of hearing thresholds following short-term or moderate noise exposure	Irreversible elevation of hearing thresholds following prolonged or intense noise exposure
Duration	Minutes to hours; typically resolves within 24–72 h	Persistent hearing loss lasting days to lifelong
Mechanism	Metabolic fatigue, stereocilia stiffness, reversible reduction in cochlear amplifier function	Structural injury: outer/inner hair cell loss, synaptopathy, stereocilia destruction, neural degeneration
Cochlear structures involved	Primarily outer hair cell dysfunction without cell death	Outer and inner hair cell death, ribbon synapse loss, cochlear nerve fiber degeneration
Otoacustic emissions (OAE)	Temporarily reduced but recover	Persistently reduced or absent
Audiometric findings	High-frequency threshold elevation that returns to baseline	Permanent high-frequency threshold elevation; may progress to affect speech frequencies
Associated symptoms	Temporary tinnitus, aural fullness, mild hyperacusis	Persistent tinnitus, distortion, difficulty hearing in noise
Typical causes	Short-term exposure to loud music, concerts, recreational noise	Repeated or continuous noise exposure (occupational or recreational), acoustic trauma
Reversibility	Fully reversible with adequate rest and protection	Irreversible; reflects permanent cochlear damage
Clinical relevance	Early warning sign of excessive noise exposure	Established noise-induced sensorineural hearing loss

**Table 2:** Summary of epidemiological evidence on hearing loss and auditory risk in adolescents and young adults.

Study/Year	Age Group	Findings (Prevalence/High-Frequency Loss/Tinnitus)	Trend Summary/Key Message
Marques et al., 2015 [37]	10–25 years (adolescents and young adults)	Social/recreational noise exposure associated with measurable hearing loss in a substantial proportion of participants; high-frequency involvement predominates.	Early NIHL already present in adolescents and young adults with leisure noise exposure, suggesting a growing public health problem.
Su & Chan, 2017 [38]	12–19 years (US adolescents)	Prevalence of hearing loss (>15 dB) increased from 17.0% (NHANES III) to 22.5% (2007–2008), then fluctuated to 15.2% (2009–2010).	Data show a notable proportion of adolescents with measurable hearing loss and periods of increased prevalence, raising concern about recreational noise and other modern exposures.
Dillard et al., 2022 [18]	12–34 years (adolescents and young adults)	Estimated 0.67–1.35 billion young people worldwide at risk of hearing loss from unsafe listening practices via personal listening devices and loud entertainment venues.	Demonstrates the massive global scale and growing burden of unsafe listening in adolescents and young adults, underscoring the need for policy action.
Haruna et al., 2023 [15]	Young adults in tertiary institutions	Hearing impairment is significantly more common in prolonged headphone/earphone users; predominantly mild, bilateral, high-frequency SNHL.	Confirms that habitual headphone/earphone use among young adults is associated with early HF-SNHL, supporting the role of recreational listening as a key risk factor.
Rabinowitz et al., 2006 [39]	Young adults entering industrial work	Over two decades, incoming young workers showed worsening baseline hearing thresholds, particularly at high frequencies.	Suggests that recreational noise (in addition to occupational factors) may have contributed to poorer hearing status in successive cohorts of young adults.
Kaur et al., 2025 [40]	Medical, nursing and pharmacy students (young adults)	High prevalence of unsafe headphone use; early auditory symptoms and measurable high-frequency threshold shifts reported in a significant subset.	Reinforces that unsafe personal listening habits in young adults are an emerging and ongoing contributor to early-onset hearing problems.
Huß et al., 2024 [41]	Young adults (17–25 years)	High prevalence of unsafe noise exposure with self-reported auditory symptoms and early OAE changes	Adds longitudinal insight into leisure noise transitions in young adults

**Table 3:** Differences between pediatric, young adult and older adult hearing loss.

Feature	Pediatric Population	Young Adults	Older Adults
Typical onset	Congenital, early childhood	Late adolescence to 30 s	Mid-life to advanced age
Primary causes	Genetic (syndromic and non-syndromic), congenital infections (CMV, rubella), otitis media, anatomical malformations	Recreational noise exposure, unsafe listening practices, personal listening devices, early occupational noise, ototoxic exposures, synaptopathy	Presbycusis (age-related), long-term metabolic degeneration, cumulative noise exposure, vascular factors
Dominant mechanism of injury	Abnormal cochlear development, genetic mutations, inflammatory damage	Outer hair cell dysfunction, early high-frequency loss, cochlear synaptopathy (“hidden hearing loss”)	Stria vascularis atrophy, reduced endocochlear potential, widespread hair cell and neural degeneration
Audiometric pattern	Flat or sloping SNHL; may include conductive components	High-frequency notch or early HF-SNHL with normal speech frequencies	Symmetric down-sloping high-frequency SNHL progressing to speech frequencies
Speech perception	Delayed language development; difficulty with speech recognition	Difficulty hearing in noise, early speech-in-noise deficits, tinnitus	Marked speech-in-noise impairment, reduced temporal processing
Reversibility/progression	Often stable but may be progressive depending on etiology	Potentially preventable; progression depends on ongoing noise exposure	Progressive and age-dependent
Associated symptoms	Developmental delay, balance issues in syndromic forms	Tinnitus, hyperacusis, temporary threshold shifts, “hidden” deficits	Poor sound discrimination, tinnitus, central auditory decline
Preventability	Limited (genetic/infectious)	High—modifiable behavioral exposures	Limited—age-related mechanisms dominate
Public health implications	Importance of screening for congenital hearing loss	Highest prevention potential; rising global burden	Large burden due to aging demographics
Diagnostic considerations	Genetic testing, newborn hearing screening	OAEs, extended high-frequency audiometry, speech-in-noise testing, synaptopathy markers	Conventional audiometry + central auditory processing evaluation

Note: Adapted from current evidence on age-specific mechanisms and epidemiology of hearing loss [11,13,16,20,63,77,88].

**Table 4:** Risk factors with main characteristics.

Risk Factor	Pathophysiology/Mechanism	Assessment Method	Prevention/Mitigation
Noise Exposure (Recreational and Occupational)	Cochlear hair cell damage, synaptopathy, hidden hearing loss	Pure-tone audiometry, Extended high-frequency audiometry (EHF 9–20 kHz), Otoacoustic emissions (OAEs), Speech-in-noise tests	Safe listening campaigns, volume limits on headphones, earplugs in loud environments, occupational noise regulations [3,4,10,14,18,20,21,22,23,26]
Genetic Susceptibility	Mutations (e.g., GJB2, mitochondrial DNA) increase vulnerability to cochlear damage and ototoxicity	Family history screening, Genetic testing in high-risk individuals	Genetic counseling, avoid excessive noise and ototoxic drugs in susceptible individuals [28,32]
Infections (Viral and Bacterial)	Cochlear inflammation, direct viral injury (e.g., SARS-CoV-2, mumps, rubella), meningitis-related cochlear damage	Audiometry, OAEs, MRI if indicated	Vaccination, prompt treatment of infections, monitoring post-infection [29,58,89,90]
Ototoxic Medications and Chemicals	Aminoglycosides, cisplatin, loop diuretics damage hair cells; recreational drugs affect cochlear vasculature; antidepressants interfere with GABA receptors in the inner ear	Baseline and serial audiometry, OAEs for early detection	Limit use of ototoxic drugs, dose adjustment, periodic monitoring, avoidance of recreational ototoxic drugs [32,33,72,76]
Lifestyle Factors (Smoking, Alcohol, Diet, Stress)	Oxidative stress, vascular compromise, reduced cochlear resilience	Audiometry, OAEs, lifestyle questionnaires	Smoking cessation, balanced diet, moderate alcohol use, stress management [33]
Psychosocial and Sociodemographic Factors	Limited awareness, peer influence, delayed care	Questionnaires, awareness surveys	Public education, school/university hearing campaigns, access to hearing care [17,18]
Aggravating Factors (Cumulative Exposure and Delayed Diagnosis)	Accelerates cochlear damage, progression from subclinical to overt hearing loss	Serial audiometry, EHF, OAEs, speech-in-noise tests	Early screening programs, combined risk mitigation strategies [9,26,51,52,89]

## Data Availability

No new data were created or analyzed in this study.
